# Multiparametric Contrast-Enhanced Ultrasound in Early Prediction of Response to Neoadjuvant Chemotherapy and Recurrence-Free Survival in Breast Cancer

**DOI:** 10.3390/diagnostics13142378

**Published:** 2023-07-14

**Authors:** Caifeng Wan, Liheng Zhou, Hongli Li, Lin Wang, Fenghua Li, Wenjin Yin, Yaohui Wang, Lixin Jiang, Jinsong Lu

**Affiliations:** 1 Department of Breast Surgery, Renji Hospital, School of Medicine, Shanghai Jiao Tong University, Pujian Rd., Shanghai 200127, China; wancaifeng@renji.com (C.W.); zhouliheng@renji.com (L.Z.); yinwenjin@renji.com (W.Y.); wangyaohui@renji.com (Y.W.); 2 Department of Ultrasound, Renji Hospital, School of Medicine, Shanghai Jiao Tong University, Pujian Rd., Shanghai 200127, China; lihongli@renji.com (H.L.); 16025@renji.com (L.W.); lifenghuaprof@sina.com (F.L.)

**Keywords:** neoadjuvant chemotherapy, breast cancer, contrast-enhanced ultrasound, pathologic complete response, recurrence-free survival

## Abstract

We aimed to explore the value of contrast-enhanced ultrasound (CEUS) in early prediction of pathologic complete response (pCR) and recurrence-free survival (RFS) in locally advanced breast cancer (LABC) patients treated with neoadjuvant chemotherapy (NAC). LABC patients who underwent CEUS before and during NAC from March 2014 to October 2018 were included and assessed. Logistic regression analysis and the Cox proportional hazards model were used to identify independent variables associated with pCR and RFS. Among 122 women, 44 underwent pCR. Molecular subtype, peak intensity (PEAK) and change in diameter were independent predictors of pCR after one cycle of NAC (area under the receiver operating characteristic curve [AUC], 0.81; 95% CI: 0.73, 0.88); Molecular subtype, PEAK and change in time to peak (TTP) were independently associated with pCR after two cycles of NAC (AUC, 0.85; 95% CI: 0.77, 0.91). A higher clinical T (hazard ratio [HR] = 4.75; 95% CI: 1.75, 12.87; *p* = 0.002) and N stages (HR = 3.39; 95% CI: 1.25, 9.19; *p* = 0.02) and a longer TTP (HR = 1.06; 95% CI: 1.01, 1.11; *p* = 0.02) at pre-NAC CEUS were independently associated with poorer RFS. CEUS can be used as a technique to predict pCR and RFS early in LABC patients treated with NAC.

## 1. Introduction

Neoadjuvant chemotherapy (NAC) has become one of the most important strategies for patients with locally advanced breast cancer (LABC), since it not only reduces primary tumor burden but also facilitates assessment of individual response at an early point [1]. Due to the high heterogeneity of breast cancer, the response to chemotherapy is different, and only a minority of patients achieve pathologic complete response (pCR) [2]. Patients achieving pCR after NAC have better disease-free survival and overall survival than those with nonpCR [3]. Therefore, early prediction of treatment response and clinical outcome with non-invasive imaging methods might play an important role in potential treatment plan modification.

In recent years, magnetic resonance imaging (MRI), positron emission tomography/computed tomography (PET/CT) and ultrasound have been investigated to evaluate tumor response to chemotherapy by depicting the changes in tumor size, metabolic activity or blood perfusion [4,5,6,7]. MRI has been considered to be the standard method for assessing NAC response in patients with breast cancer [8,9]. The sensitivity of MRI in correctly identifying residual breast cancer ranges from 83% to 92%, but the specificity was relatively low, ranging from 54% to 68% [10,11,12,13]. MRI could overestimate or underestimate the extent of the residual breast tumor due to the difficulties in interpreting extravascular and extracellular volume changes [14,15]. Repeated gadolinium-based contrast agent administration is associated with neuronal tissue deposition in the setting of relatively normal renal function [16] and might affect the permeability of the blood–brain barrier [17]. In addition, patients with claustrophobia, metal implants, pacemakers or insulin pumps could not undergo contrast-enhanced MRI examination.

Contrast-enhanced ultrasound (CEUS) can facilitate the dynamic observation and quantification of tumor perfusion [18] and may have the ability to differentiate between non-vascularized therapy-induced fibrosis and residual tumors without exposing the patients to any risk of radiation. Several studies have investigated the value of CEUS in evaluating tumor perfusion after chemotherapy, and the preliminary results were promising [19,20,21,22]. However, they had relatively small study populations, and there was no consensus on the predictive value of CEUS at different time-points during NAC in LABC patients. In addition, the value of CEUS in predicting the recurrence-free survival (RFS) of LABC patients after NAC has not been assessed. The purpose of this study was to investigate the optimal parameters of CEUS for early prediction of pCR and RFS in LABC patients treated with NAC. 

## 2. Materials and Methods 

### 2.1. Patients 

This retrospective interpretation of prospective data study was approved by the institutional ethics committee. Written informed consent was obtained from all patients. From March 2014 to October 2018, consecutive women aged 18 to 70 years with histologically confirmed LABC who underwent CEUS examination pretreatment and after one or two cycles of NAC were considered for inclusion. Exclusion criteria were unavailability of CEUS images pretreatment or after one and two cycles of NAC, uncompleted full cycles of NAC and no surgery after chemotherapy. Patients included in this study were from two separately registered clinical trials (SHPD001 [NCT02199418] and SHPD002 [NCT02221999]). The protocols of the study were published previously [23]. All patients received paclitaxel-cisplatin-based NAC. In brief, cisplatin (25 mg/m^2^) on day 1, 8, and 15 every 28 days combined with paclitaxel (80 mg/m^2^) on day 1, 8, 15, and 22 for 4 cycles. In some patients with HER2/receptor-positive lesions, concomitant trastuzumab was used in combination with the chemotherapy regimen on a weekly basis (the first dose, 4 mg/kg; the subsequent dose, 2 mg/kg on day 1, weekly for 16 weeks.). Patients with hormone receptor-positive breast cancer in SHPD002 were randomized to chemotherapy combined with endocrine therapy on the basis of their menstrual status or chemotherapy alone. In SHPD002, premenopausal patients with triple-negative breast cancer were randomized to chemotherapy with or without suppression of ovarian function. Surgery was performed within one week after the final contrast-enhanced ultrasound (CEUS) examination. The final CEUS examination was performed at least 2 days after the final chemotherapy.

### 2.2. CEUS Examination and Image Analysis 

The ultrasonography examination in 2D B-mode was performed using MyLab Twice (Esaote, Genoa, Italy) with a 4–13-MHz LA523 linear transducer. Color Doppler US was performed to dynamically observe the whole lesion, and the plane with the richest vessels was identified by two board-certified breast radiologists for CEUS (C.W. and L.W. both with 8 years of experience in breast CEUS). If uncertainty occurred, another board-certified breast radiologist (F.L., with more than 10 years of experience in breast CEUS) reviewed the image until consensus was achieved. The selected plane for CEUS should, if possible, include the lesion and its surrounding normal tissue. CEUS was performed with the MyLab Twice unit and a 4.5–7.5-MHz LA332 linear transducer. Contrast-tuned imaging and microflow imaging software were incorporated in the system. SonoVue (Bracco, Milan, Italy) was used as the contrast agent. The injection method and dose were recommended by the manufacturer. For CEUS examination, the machine parameters were adjusted as follows: frequency, general-M mode; gain, 45%; derated pressure, 50 kPa (expressing a mechanical index of 0.06). A volume of 2.5 mL (for contrast-tuned imaging) of SonoVue was injected via the antecubital vein in a bolus fashion through a 20-gauge intravenous cannula within 1–2 s, followed by a flush with 5 mL of 0.9% normal saline solution. Continuous imaging was performed immediately after injection of the SonoVue and lasted for 6 min. The quantitative acquisition time for kinetic analysis was 3 min. After a waiting period of 15 min, 2.4 mL of the contrast agent was administrated for microflow imaging for qualitative analysis. According to the guidelines of the American Joint Committee on Cancer, the assessment was always performed in the same single tumor if the patient had multicentric or multifocal diseases. In this study, only the lesion with the largest diameter was evaluated in patients with multiple lesions.

The presence or absence of perfusion defect and radial or penetrating vessels at pre-NAC CEUS were recorded [18]. Quantitative parameters of the time-intensity curve (TIC) were obtained with a specific sonographic quantification software (Qontrast, Bracco, Milan, Italy). The region of interest (ROI) was defined manually around the whole lesion. Areas of calcifications, necrosis and ligaments were avoided. For quantitative analysis, the software generated a gamma-varied time-intensity curve (TIC) based on the area of region of interest (ROI). Quantitative parameters were categorized as follows: PEAK: the maximum intensity of the TIC during bolus transit. RBV (regional blood volume): total blood volume in the ROI region, proportionate to the area under the curve. TTP (s) (time to peak): time needed to reach peak intensity, beginning from the time the first microbubble reached the lesion. RBF (regional blood flow, RBV/MTT): relative blood flow in the area under investigation. MTT (s) (mean transit time): circulation time of contrast agent in the ROI region. CEUS image analysis was performed in a blinded manner by two board-certified radiologists (C.W. and L.W., both with 8 years of experience in breast CEUS) without clinical-pathologic information being made available. If disagreement occurred, another board-certified radiologist (H.L., with 10 years of experience in breast CEUS) reviewed the images until consensus was achieved. Surgery was performed within one week after the final contrast-enhanced ultrasound (CEUS) examination. The final CEUS examination was performed at least 2 days after the final chemotherapy.

### 2.3. Clinical-Pathologic Evaluation and Follow-Up

Pathologic response was assessed by two pathologists with more than 15 years of experience in breast pathologic analysis. For this study, pCR was defined as no residual invasive cancer in breast tissue. Lymph node status was not included. Patient’s age, clinical T and N stages and NAC regimen were collected. Pathologic data on human epidermal growth factor receptor 2 (HER2), Ki-67 status, estrogen receptor (ER) and progesterone receptor (PR) were collected from reports of biopsies performed prechemotherapy. ER and PR were considered positive if 1% or more of tumor cells had nuclear staining with any intensity. HER2 expression was evaluated by using fluorescence in situ hybridization. Expression of HER2 was considered positive if the immuno-histochemistry 3+ or the ratio of HER2 gene signals to chromosome 17 signals >2.0 or HER2 gene copy >6.0. Ki-67 expression was assessed by determining the number of positive stained nuclei, with at least 14% of stained cells indicating high-level expression. Tumor subtypes were categorized according to the St. Gallen Consensus [24]: luminal A-like (ER-positive and/or PR-positive, HER2-negative, and Ki-67 expression <14%), luminal B-like (ER-positive and/or PR-positive; HER2-positive/negative and Ki-67 expression ⩾14%), HER2-enriched (ER and PR-negative, and HER2-positive) and triple-negative (ER-negative, PR-negative, and HER2-negative).

After surgery, all patients were followed up through physical examinations, mammography, ultrasonography and MRI. Abdomen and chest computed tomography (CT), bone scans and fuorine-18-fuorodeoxyglucose-combined PET-CT were also performed if needed. The date when the recurrent lesion was diagnosed by imaging or pathologic examination was identified as the date of recurrence. In patients who did not develop recurrence, this was defined as the interval between the date of surgery and the last follow-up. Patients who did not develop recurrence at the last follow-up date were treated as censored observations. Patients who were lost to follow-up were excluded in this part. Assessment of recurrence was assessed by one breast surgeon with more than 12 years of experience in the diagnosis and treatment of breast diseases.

### 2.4. Statistical Analysis 

Continuous variables were expressed as mean ± standard deviation. Categorical variables were expressed as numbers. The Shapiro–Wilk test was used to test the normal distribution of continuous variables. The Levene F test was used to test the equal variance. Categorical variables were evaluated with either the Pearson *X^2^* test or Fisher’s exact test when the expected value in any cell was less than 5. For the continuous variables, if the data were normally distributed and had equal variance, the Student’s t-test was used. Otherwise, the Mann–Whitney U test was performed. Changes in the quantitative parameters of CEUS and tumor diameter during NAC were calculated with the following formula:

$$\Delta A=\frac{value\;after\;NAC-value\;before\;NAC}{value\;before\;NAC}\times 100\%$$

To test the independence of established factors in predicting pCR, multivariable logistic regression analysis was used. The diagnostic performance in differentiating between pCR and nonpCR patients was assessed by using the receiver operating characteristic curve (ROC). 

RFS was defined as the time from surgery to the date of the first recurrence, including local-regional recurrence and distant metastasis. The Cox proportional hazards model was used to determine the associations of the clinical-pathologic variables and parameters obtained at pre-NAC CEUS with RFS. To dichotomize quantitative parameters of CEUS for survival analysis, the optimal cutoff values were determined by using the maximum Youden index with ROC analysis. Survival curves were estimated through Kaplan–Meier analysis, and statistically significances were examined with log-rank tests. 

To test the independence of molecular subtype to predict pCR and RFS, it was classified into two groups: luminal or HER2-enriched and triple-negative. Variables with *p* < 0.1 at univariate analysis were included in the multivariable analysis through the forward stepwise selection method. *p* < 0.05 was considered to indicate a significant difference. Our statistical analyses were performed using IBM SPSS Statistics 22 (Armonk, NY, USA). The ROC curve analysis was performed using MedCalc for Windows (version 11.2.1.0; MedCalc Software, Mariakerke, Belgium).

## 3. Results

### 3.1. Patient Characteristics 

**Figure 1:**
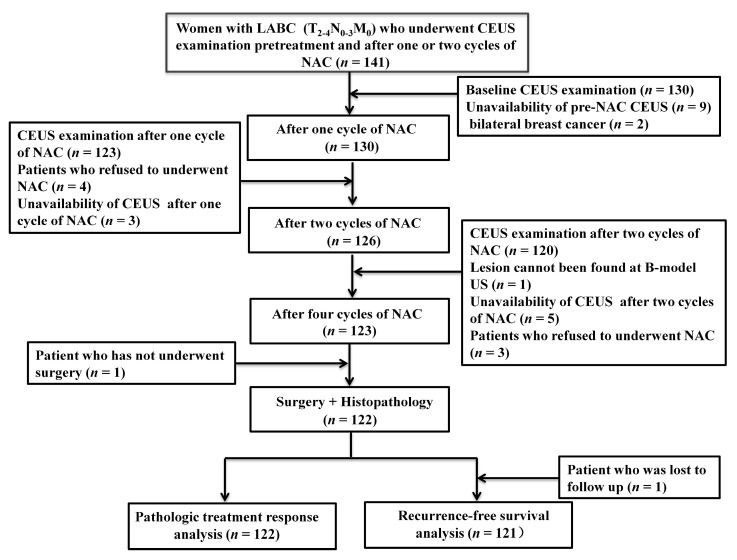
Flow chart of this study. LABC = locally advanced breast cancer. CEUS = contrast-enhanced ultrasound. NAC = neoadjuvant chemotherapy.

A total of 139 consecutive women with histologically confirmed LABC who underwent CEUS examination pretreatment and after one or two cycles of NAC were considered for inclusion. Of the 139 patients, 17 were excluded owing to unavailability of CEUS images pretreatment or after one and two cycles of NAC (*n* = 9), uncompleted study treatment (*n* = 7) or no surgery after chemotherapy (*n* = 1). Accordingly, 122 women comprised the study group for pathologic treatment response analysis. One patient was lost to follow-up, and 121 patients were included for recurrence-free survival analysis (Figure 1).

### 3.2. Pathologic Treatment Response

Characteristics were described and compared between the pCR and nonpCR groups (Table 1). The tumor diameter ranged from 1.4 to 10 cm (mean, 4.2 cm) prechemotherapy. All patients received modified radical mastectomy. A therapeutic response was obtained in 44 (36.1%, 44/122) patients with pCR. ER (*p* < 0.001) and PR (*p* < 0.001) receptor negativity, HER2 receptor positivity (*p* < 0.001) and high Ki-67 index (*p* = 0.004) were associated with pCR. Molecular subtypes of triple negative and HER2-enriched were associated with pCR (*p* < 0.001). Of the 44 patients with pCR, 29 patients showed no evidence of malignant cells in the breast and sampled axillary lymph nodes. Thirty-eight patients showed no evidence of malignant cells in the breast and six patients showed ductal carcinoma in situ only. Partial response was observed in 75 patients. Twenty-two patients showed only a few scattered tumor cells remained or that the residual tumor was less than 0.5 cm in size in the breast. Stable diseases were observed in three patients. No patient showed progression disease.

### 3.3. Factors Associated with PCR 

The pCR and nonpCR groups were comparable for the presence of radial or penetrating vessels (*p* = 0.50), PEAK (*p* = 0.46), TTP (*p* = 0.19), RBV (*p* = 0.25), RBF (*p* = 0.60) and MTT (*p* = 0.06) at pre-NAC CEUS. Perfusion defect was seen in 46 lesions in the non-pCR group before NAC, which showed a significantly higher than that in pCR group (*p* = 0.02). (Table 2).

**Figure 2:**
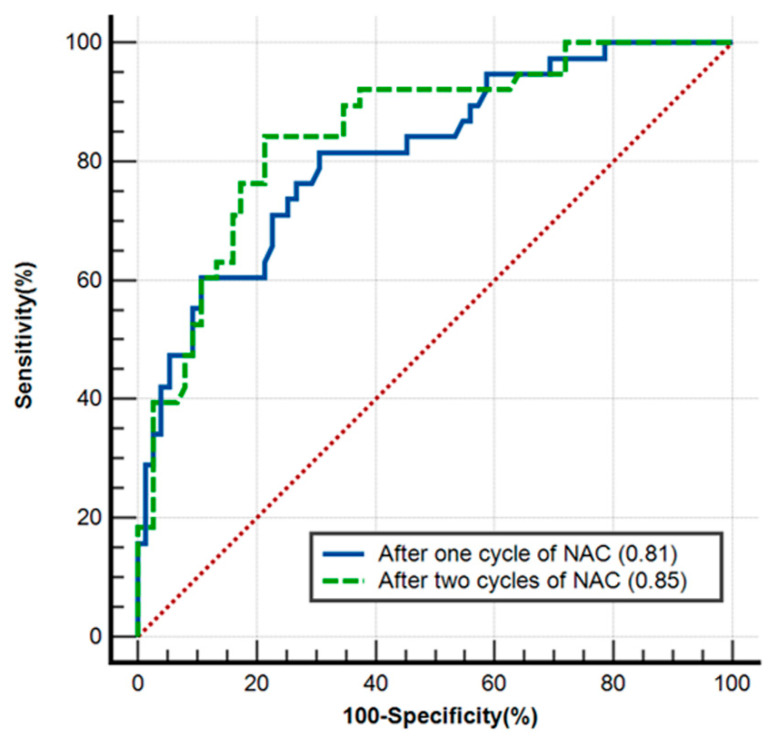
Receiver operating characteristic curve (ROC) of the after one and two cycles of neoadjuvant chemotherapy (NAC) models. After one cycle of NAC, the area under the ROC (AUC) for the combination of molecular subtype, peak intensity (PEAK_1_) and change in diameter (Δdiameter_1_) was 0.81 (Az1; 95% CI: 0.73, 0.88) (blue solid line). After two cycles of NAC, the AUC for the combination of molecular subtype, PEAK_2_ and change in time to peak (ΔTTP_2_) was 0.85 (Az2; 95% CI: 0.77, 0.91) (green dashed line). No significant difference was found between the values of Az1 and Az2 (*p* = 0.39).

After one cycle of NAC, PEAK_1_, RBV_1_, RBF_1_, diameter_1_ and ΔPEAK_1_, ΔTTP_1_, ΔRBF_1_ and Δdiameter_1_ were associated with pCR (all, *p* < 0.05) (Table 2). Multivariable logistic regression analysis showed that molecular subtype (odds ratio [OR], 6.13; 95% confidence interval [CI]: 2.05, 18.36; *p* = 0.001), PEAK_1_ (OR, 1.05; 95% CI: 1.02, 1.09; *p* = 0.004) and Δdiameter_1_ (OR, 0.004; 95% CI: 0.00, 0.16; *p* = 0.003) were independently associated with pCR (Table 3). The area under the ROC (AUC) for the combination of them (Az1) to predict pCR was 0.81 (95% CI: 0.73, 0.88), and the sensitivity and specificity to predict pCR were 74.4% and 73.2%, respectively (Figure 2).

After two cycles of NAC, PEAK_2_, TTP_2_, RBV_2_, RBF_2_, diameter_2_, ΔPEAK_2_, ΔTTP_2_, ΔRBF_2_, MTT_2_ and Δdiameter_2_ were associated with pCR (all, *p* < 0.05) (Table 2). Multivariable logistic regression analysis showed that molecular subtype (OR, 4.09; 95% CI: 1.31, 12.77; *p* = 0.02), PEAK_2_ (OR, 1.098; 95% CI: 1.05, 1.15; *p* < 0.001) and ΔTTP_2_ (OR, 0.47; 95% CI: 0.23, 0.94; *p* = 0.03) were independently associated with pCR (Table 3). The AUC for the combination of them (Az2) to predict pCR was 0.85 (95% CI: 0.77, 0.91) (Figure 2), and the sensitivity and specificity to predict pCR were 78.7% and 85.4%, respectively. No significant difference was found between the values of Az1 and Az2 (*p* = 0.39).

### 3.4. Recurrence Outcome

The median follow-up period was 63 months (range, 12–92 months). A total of 17 (14.05%, 17/121) recurrences developed. Of the 17 patients with recurrence, 14 (82.35%, 14/17) underwent nonpCR. The sites of metastases include bones (*n* = 5), lung (*n* = 5), brain (*n* = 4), liver (*n* = 2) and contralateral axillary (*n* = 1). The recurrence and nonrecurrence groups differed for the clinical T (*p* < 0.001) and N stages (*p* < 0.001) and the use of adjuvant endocrine therapy (*p* = 0.045) (Table 1). With regard to the parameters at pre-NAC CEUS, the TTP value of the recurrence group was significantly higher than that of the nonrecurrence group (36.67 ± 8.20 vs. 29.86 ± 9.26, *p* = 0.005). Perfusion defect was more frequently observed in the recurrence group (*p* = 0.006) (Table 4).

### 3.5. Univariate and Multivariate Cox Proportional Hazards Analyses

**Figure 3:**
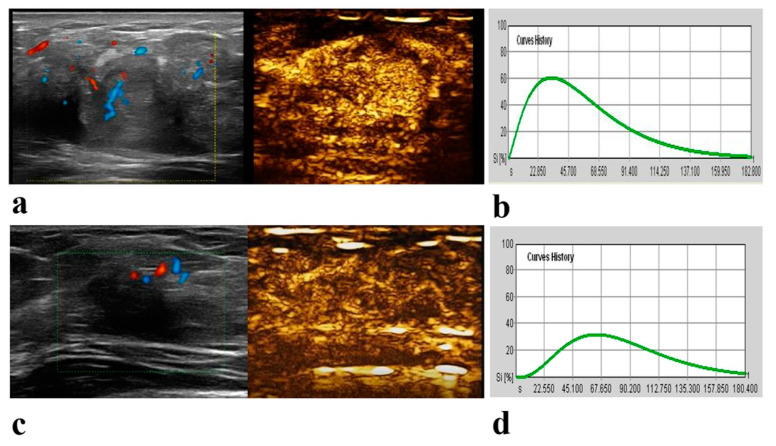
A 33-year-old woman with 4.0 cm triple-negative breast cancer of the right breast following neoadjuvant chemotherapy (NAC). Before NAC, Color Doppler US and contrast-enhanced ultrasound (CEUS) images obtained 38 s after contrast agent injection show an inhomogeneous hyper-enhancement lesion with an indistinct margin (**a**). The time-intensity curve (TIC) shows peak intensity (PEAK) is 61.70; time to peak (TTP), 32.26 s; regional blood volume (RBV), 4834.48; regional blood flow (RBF), 81.67, and mean transit time (MTT), 59.20 s. (**b**). After two cycles of NAC, Color Doppler US and CEUS images obtained 59 s after contrast agent injection show an inhomogeneous hyper-enhancement lesion with an indistinct margin (**c**). The TIC shows PEAK is 32.60; TTP, 63.19 s; RBV, 3271.80; RBF, 38.22, and MTT, 85.61 s (**d**). After surgery, pathological analysis found no residual cancer in the breast and sampled axillary lymph node. During 85 months of follow-up, there was no evidence of recurrence.

**Figure 4:**
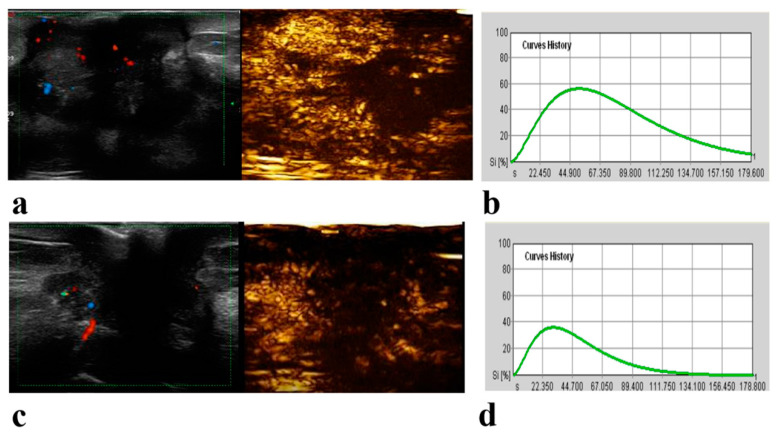
A 56-year-old woman with 4.6 cm luminal B breast cancer of the left breast following neoadjuvant chemotherapy (NAC). Before NAC, Color Doppler US and contrast-enhanced US (CEUS) images obtained 54 s after contrast agent injection show a hyper-enhanced lesion with local large patchy perfusion defect and an indistinct margin (**a**). Time-intensity curve (TIC) analysis shows peak intensity (PEAK) is 57.60; time to peak (TTP), 50.90 s; regional blood volume (RBV), 6041.92; regional blood flow (RBF), 72.75, and mean transit time (MTT), 83.05 s (**b**). After two cycles of NAC, Color Doppler US and CEUS images obtained 35 s after contrast agent injection show an iso-enhanced lesion with local large patchy perfusion defect and an indistinct margin (**c**). The TIC shows PEAK is 37; TTP, 29.95 s; RBV, 2360.43; RBF, 46.84, and MTT, 50.39 s (**d**). After surgery, pathological analysis showed only a few scattered tumor cells remained in the breast. This patient was found to have contralateral axillary metastasis after a follow-up of 67 months.

According to the univariate Cox regression analysis, a higher clinical T stage (hazard ratio [HR] = 5.38; 95% CI: 1.99, 14.57; *p* = 0.001) and N stage (HR = 3.495; 95% CI: 1.29, 9.49; *p* = 0.01), the presence of perfusion defect (HR = 0.20; 95% CI: 0.06, 0.71; *p* = 0.01) and a longer TTP (HR = 1.06; 95% CI: 1.02, 1.095; *p* = 0.004) at pre-NAC CEUS were associated with poorer RFS (Table 5). In the multivariable Cox proportional hazards analysis, variables associated with worse RFS were a higher clinical T stage (HR = 4.75; 95% CI: 1.75, 12.87; p = 0.002) and N stage (HR = 3.39; 95% CI: 1.25, 9.19; *p* = 0.02), and a longer TTP (HR = 1.06; 95% CI: 1.01, 1.11; *p* = 0.02) at pre-NAC CEUS (Table 5). Representative examples of pCR and nonpCR lesions and recurrence and nonrecurrence lesions are given in Figure 3 and Figure 4.

### 3.6. Disease-Free Survival Analysis

**Figure 5:**
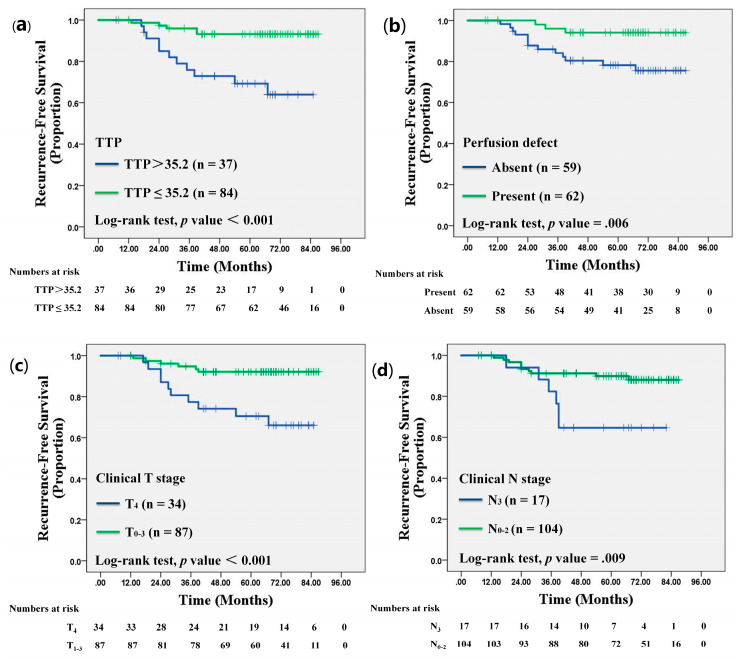
Kaplan–Meier survival curves for recurrence-free survival according to: (**a**) time to peak obtained at contrast-enhanced US (CEUS) before neoadjuvant chemotherapy (NAC), (**b**) perfusion defect obtained at CEUS before NAC, (**c**) clinical T stage prior to NAC, and (**d**) clinical N stage prior to NAC in 121 women with locally advanced breast cancer.

According to ROC curve analysis, the optimal cutoff value for TTP to assess an association with RFS was 35.2 (sensitivity, 68.8%; specificity, 74.2%; AUC: 0.73; 95% CI: 0.64, 0.80). Kaplan–Meier survival analysis showed that patients who had tumors exhibiting a longer TTP (>35.2) and perfusion defect at pre-NAC CEUS were significantly associated with worse RFS (Log-rank *p* < 0.001 and *p* = 0.006, respectively) (Figure 5a,b). For the clinical-pathologic variables, the Kaplan–Meier survival analysis demonstrated that a higher clinical T stage and N stage were significantly associated with worse RFS (Log-rank *p* < 0.001 and *p* = 0.009, respectively) (Figure 5c,d).

## 4. Discussion 

Due to the high heterogeneity of breast cancer, it has differential response rates to NAC and varying RFS. Identifying patients with a favorable response to NAC and a good prognosis at an early time-point has naturally become a priority in breast cancer research. Our study showed that multiparametric CEUS combined with clinical-pathologic variables can be used to predict response to chemotherapy as early as after one cycle of NAC (AUC, 0.81 and 0.85 in the after one and two cycles of NAC models, respectively). A TTP (hazard ratio [HR] = 1.056; *p* = 0.026) at pre-NAC CEUS was independently associated with poorer RFS. To our knowledge, this is the first study to evaluate the value of CEUS in predicting the RFS of LABC patients after NAC. 

Angiogenesis, which is an important index of the biological activity of the tumor, plays a crucial role in tumor growth and metastasis. The enhancement degree and patterns of CEUS were correlated with the density and distribution of the microvessel [18]. Therefore, it seems plausible that there are associations between chemotherapy response or clinical outcomes and the CEUS features in LABC patients treated with NAC. Quantitative analysis of tumor blood perfusion with CEUS can be used for noninvasive assessment of functional changes in tumors after chemotherapy [19,20,25,26]. Huang et al. [20] used CEUS for quantitative evaluation of breast cancer response to chemotherapy and demonstrated that changes in maximum intensity were significantly associated with good therapeutic responses after two cycles of NAC. Our results strengthened the evidence and showed that PEAK was independently associated with pCR, even after one cycle of NAC. The pathophysiologic basis for the changes in CEUS quantitative parameters may be that successful chemotherapy caused cytotoxic tumor cell death, resulting in a reduction in tissue vascular endothelial growth factor level and, hence, apoptosis of endothelial cells in immature vessels [27]. 

TTP, as a quantitative parameter of CEUS, was suggested to be an independent predictive factor of pCR [20]. Our results showed that ΔTTP is not only associated with pCR after one and two cycles of NAC, but also independently associated with pCR after two cycles of NAC. Similar to us, Sharma et al. [28] reported that after one, two and three cycles of NAC, the difference in the ΔTTP between good and poor responders was significant. But, in this study, pathological response was graded according to the Miller–Payne system of five grades. For all we know, the relationship between CEUS features and RFS has not yet been reported. In our study, patients with a longer TTP at pre-NAC CEUS were independently associated with worse RFS. Dietzel et al. [29] used semi-quantitative MRI analysis-dedicated CAD software in breast MRI to predict disease-related death in primary breast cancer patients and demonstrated that TTP was an independent predictor of survival, which may indirectly support our results.

Most of the present studies mainly focused on the value of CEUS for quantitative evaluation of tumor response to chemotherapy [19,20,25,26]. The value of the qualitative parameters of CEUS for evaluating the efficacy of NAC and RFS has not been fully assessed. With the rapid growth of malignant tumors, the vascular formation and nutrition supply are relatively insufficient. Thus, hypoxic and necrotic conditions are often found in the center of the lesions. Perfusion defect is one of the most important enhancement patterns in the differential diagnosis of breast tumors [30]. A previous study has shown that perfusion defect was associated with prognostic factors indicative of tumor aggressiveness and may be a manifestation of tumor heterogeneity [18]. In this study, perfusion defect was associated, albeit not independently, with nonpCR and worse RFS. One possible explanation is that necrotic areas in tumors usually exhibit poor perfusion and are characterized by acidity and hypoxia, which may reduce the delivery efficiency of the chemotherapy drugs [31] and may result in a higher risk of developing metastases. Tissues near necrotic regions are more likely to remain hypoxic and have slower metabolisms; thus, the chemosensitivity may be reduced [32]. 

Our study still had limitations. First, this was a single-center study, and selection bias might have occurred, mainly because patients without CEUS were excluded. Second, in this study, pCR was defined as no residual invasive cancer in breast tissue without considering axillary status. Third, due to the limited amount of data and relatively short follow-up period, it was not fit for performing an overall survival analysis.

## 5. Conclusions

In summary, our study revealed that CEUS can provide useful information about the blood perfusion of the tumor and has the potential to serve as a noninvasively functional technique for early prediction of pCR as well as RFS in LABC patients treated with NAC. However, our findings should be considered as preliminary results, and further large-scale multicentric studies with longer follow-up durations are needed to confirm our findings. 

## Figures and Tables

**Table 1:** Patient characteristics according to breast cancer pathologic response and recurrence status.

Characteristic	pCR Status			Recurrence Status		
	pCR	NonpCR	*p* Value	Recurrence	Nonrecurrence	*p* Value
Number of lesions	44 (36)	78 (64)		17 (14)	104 (86)	
Age (year) *			0.59			0.64
≤50	17 (39)	34 (44)		6 (35)	43 (41)	
>50	27 (61)	44 (56)		11 (65)	61 (59)	
Clinical T stage			0.17			<0.001
T_1–3_	35 (80)	53 (68)		6 (35)	81 (78)	
T_4_	9 (20)	25 (32)		11 (65)	23 (22)	
Clinical N stage			0.29			0.007
N_0-2_	40 (91)	64 (82)		11 (65)	93 (89)	
N_3_	4 (9)	14 (18)		6 (35)	11 (11)	
ER status			<0.001			1
ER negative	27 (61)	14 (18)		5 (29)	34 (33)	
ER positive	17 (39)	64 (82)		12 (71)	70 (67)	
PR status			<0.001			0.78
PR negative	21 (48)	12 (15)		4 (24)	30 (29)	
PR positive	23 (52)	66 (85)		13 (76)	74 (71)	
HER2 status			<0.001			0.45
HER2 negative	15 (34)	54 (69)		11 (65)	57 (55)	
HER2 positive	29 (66)	24 (31)		6 (35)	47 (45)	
Ki-67 index			0.004			0.11
<14%	0 (0)	13 (17)		13 (77)	94 (90)	
≥14%	44 (100)	65 (83)		4 (23)	10 (10)	
Molecular subtype			<0.001			0.43
Luminal A-like	0 (0)	11 (14)		3 (18)	6 (6)	
Luminal B-like (HER2 positive)	16 (36)	20 (26)		4 (24)	32 (31)	
Luminal B-like (HER2 negative)	11 (25)	39 (50)		8 (47)	43 (41)	
Triple negative	3 (7)	4 (5)		0 (0)	6 (6)	
HER2-enriched	14 (32)	4 (5)		2 (12)	17 (16)	
Adjuvant endocrine therapy			0.68			0.045
No	24 (55)	37 (47)		12 (71)	48 (46)	
Aromatase inhibitors	10 (23)	23 (30)		5 (29)	28 (27)	
Ovarian function suppression	10 (23)	18 (23)		0 (0)	28 (27)	
Adjuvant Herceptin			<0.001			0.1
Yes	23 (52)	16 (21)		2 (12)	37 (36)	
No	21 (48)	62 (80)		15 (88)	67 (64)	
Tumor diameter (cm)			0.22			0.3
Pre-treatment *	3.96 ± 1.67	4.34 ± 1.59		4.63 ± 1.93	4.10 ± 1.54	

Note. Data are number of patients and data in parentheses are percentages. pCR = pathologic complete response, ER = estrogen receptor, PR = progesterone receptor, HER2 = human epidermal growth factor receptor 2, luminal A-like (ER-positive and/or PR-positive, HER2-negative, and Ki-67 expression <14%), luminal B-like/HER2-positive (ER-positive and/or PR-positive; HER2-positive and Ki-67 expression ⩾14%), luminal B-like/HER2-negative (ER-positive and/or PR-positive; HER2-negative and Ki-67 expression ⩾14%), HER2-enriched (ER and PR-negative, and HER2-positive), and triple-negative (ER-negative, PR-negative, and HER2-negative).* Data are means ± standard deviations.

**Table 2:** CEUS parameters and tumor diameter pretreatment and during NAC between the pCR and nonpCR Groups.

Parameter	pCR	NonpCR	Total Number	*p* Value
Qualitative parameters pre-NAC				
Perfusion defect *				0.02
Present	16 (36)	46 (59)	62 (50)	
Absent	28 (64)	32 (41)	60 (50)	
Radial or penetrating vessels *				0.50
Present	17 (39)	35 (45)	52 (43)	
Absent	27 (61)	43 (55)	70 (57)	
Quantitative parameters before and during NAC				
PEAK_0_	57.75 ± 13.79	59.54 ± 12.15		0.46
TTP_0_	29.22 ± 8.06	31.78 ± 9.98		0.19
RBV_0_	5487.63 ± 2323.33	5961.44 ± 2075.39		0.25
RBF_0_	77.34 ± 19.58	79.16 ± 17.17		0.60
MTT_0_	68.53 ± 13.96	73.99 ± 15.63		0.06
PEAK_1_	45.0 ± 11.51	54.59 ± 14.44		<0.001
TTP_1_	35.04 ± 15.85	30.68 ± 9.31		0.18
RBV_1_	3833.54 ± 1915.79	4818.29 ± 2220.50		0.009
RBF_1_	58.47 ± 16.75	72.04 ± 21.41		0.001
MTT_1_	64.39 ± 22.39	65.24 ± 16.34		0.39
ΔPEAK_1_	−0.20 ± 0.22	−0.07 ± 0.22		0.002
ΔTTP_1_	0.28 ± 0.64	0.03 ± 0.39		0.02
ΔRBV_1_	−0.26 ± 0.35	−0.13 ± 0.41		0.10
ΔRBF_1_	−0.23 ± 0.24	−0.07 ± 0.26		0.002
ΔMTT_1_	−0.04 ± 0.34	−0.09 ± 0.26		0.42
PEAK_2_	30.91 ± 9.40	45.90 ± 13.79		<0.001
TTP_2_	44.52 ± 19.93	35.44 ± 12.67		0.02
RBV_2_	2783.98 ± 1278.59	4057.81 ± 2072.23		<0.001
RBF_2_	39.26 ± 15.53	59.40 ± 19.62		<0.001
MTT_2_	72.16 ± 22.12	66.47 ± 16.98		0.23
ΔPEAK_2_	−0.43 ± 0.24	−0.22 ± 0.27		<0.001
ΔTTP_2_	0.68 ± 1.03	0.20 ± 0.533		0.004
ΔRBV_2_	−0.41 ± 0.35	−0.27 ± 0.44		0.08
ΔRBF_2_	−0.46 ± 0.26	−0.24 ± 0.28		<0.001
ΔMTT_2_	0.11 ± 0.41	−0.07 ± 0.31		0.01
Tumor diameter during NAC				
Diameter_1_	25.93 ± 10.88	33.34 ± 13.39		0.002
ΔDiameter_1_	−0.32 ± 0.13	−0.23 ± 0.12		0.001
Diameter_2_	20.70 ± 10.49	27.61 ± 12.99		0.005
ΔDiameter_2_	−0.48 ± 0.19	−0.37 ± 0.15		0.001

Note. Data are means ± standard deviations. CEUS = contrast-enhanced ultrasound, NAC = neoadjuvant chemotherapy, pCR = pathologic complete response, PEAK = peak intensity, TTP = time to peak, RBV = regional blood volume, RBF = regional blood flow, MTT = mean transit time. The quantitative parameters of CEUS and tumor diameter pretreatment and after one and two cycles of NAC were described as A_0,_ A_1_ and A_2._ The relative changes in CEUS quantitative parameters and tumor diameter after one and two cycles of NAC were describe as ΔA_1_ and ΔA_2_ and were calculated with the following formula: ΔA = ([value after NAC-value before NAC]/value before NAC) × 100%. * Data are number of patients and data in parentheses are percentages.

**Table 3:** Multivariable logistic regression analyses of CEUS and clinical-pathologic variables associated with pCR.

Variable	Beta Coefficient	Odds Ratio	95% CI	*p* Value
After one cycle of NAC				
PEAK_1_	0.052	1.05	1.02, 1.09	0.004
ΔDiameter_1_	−5.541	0.004	0.00, 0.16	0.003
Molecular subtype	1.813	6.13	2.05, 18.36	0.001
After two cycles of NAC				
PEAK_2_	0.094	1.098	1.05, 1.15	<0.001
ΔTTP_2_	−0.76	0.47	0.23, 0.94	0.03
Molecular subtype	1.409	4.09	1.31, 12.77	0.02

Note. CEUS = contrast-enhanced ultrasound, pCR = pathologic complete response, CI = confidence interval, NAC = neoadjuvant chemotherapy, PEAK = peak intensity, TTP = time to peak. The quantitative parameters of CEUS and tumor diameter after one and two cycles of NAC were described as A_1_ and A_2._ The relative changes in CEUS quantitative parameters and tumor diameter after one and two cycles of NAC were described as ΔA_1_ and ΔA_2_ and were calculated with the following formula: ΔA = ([value after NAC—value before NAC]/value before NAC) × 100%.

**Table 4:** Quantitative and qualitative parameters of CEUS before NAC between the recurrence and nonrecurrence groups.

Parameter	Recurrence	Nonrecurrence	Total Number	*p* Value
Quantitative parameters				
PEAK_0_	58.52 ± 10.71	59.11 ± 12.99		0.86
TTP_0_	36.67 ± 8.20	29.86 ± 9.26		0.005
RBV_0_	5525.26 ± 1636.11	5807.69 ± 2243.84		0.62
RBF_0_	75.95 ± 16.57	78.84 ± 18.35		0.54
MTT_0_	71.52 ± 12.51	71.85 ± 15.52		0.93
Qualitative parameters				
Perfusion defect				0.006
Present	14 (82)	48 (46)	62 (51)	
Absent	3 (18)	56 (54)	59 (49)	
Radial or penetrating vessels				0.50
Present	6 (35)	46 (44)	52 (43)	
Absent	11 (65)	58 (56)	69 (57)	

Note. CEUS = contrast-enhanced ultrasound, NAC = neoadjuvant chemotherapy, PEAK = peak intensity, TTP = time to peak, RBV = regional blood volume, RBF = regional blood flow, MTT = mean transit time. The quantitative parameters of CEUS pretreatment were described as A_0_.

**Table 5:** Univariable and multivariable Cox proportional hazards analyses of variables associated with RFS.

Characteristic	Univariable Analysis			Multivariable Analysis		
	Hazard Ratio	95% CI	*p* Value	Hazard Ratio	95% CI	*p* Value
Age (year)	0.99	0.95, 1.03	0.53			
Clinical T stage			0.001			0.002
T_1~3_	Reference category			Reference category		
T_4_	5.38	1.99, 14.57		4.75	1.75, 12.87	
Clinical N stage			0.01			0.02
N_0~2_	Reference category			Reference category		
N_3_	3.495	1.29, 9.49		3.39	1.25, 9.19	
ER status			0.87			
ER negative	Reference category					
ER positive	0.92	0.32, 2.60				
PR status			0.76			
PR negative	Reference category					
PR positive	0.84	0.27, 2.58				
HER2 status			0.51			
HER2 negative	Reference category					
HER2 positive	1.395	0.52, 3.77				
Ki-67 index			0.12			
>14	2.42	0.79, 7.43				
≤14	Reference category					
Molecular subtype			0.39			
Luminal	Reference category					
Triple negative and HER2-enriched	0.53	0.12, 2.31				
Adjuvant endocrine therapy			0.05			
Yes	0.35	0.12, 0.995				
No	Reference category					
Adjuvant Herceptin			0.08			
Yes	Reference category					
No	0.27	0.06, 1.19				
Tumor diameter	1.02	0.99, 1.05	0.19			
CEUS parameters pre-NAC						
perfusion defect			0.01			
Present	0.20	0.06, 0.71				
Absent	Reference category					
Radial or penetrating vessels			0.50			
Present	1.41	0.52, 3.81				
Absent	Reference category					
PEAK_0_	0.99	0.96, 1.03	0.82			
TTP_0_	1.06	1.02, 1.095	0.004	1.06	1.01, 1.11	0.02
RBV_0_	1.00	1.00, 1.00	0.60			
RBF_0_	0.99	0.97, 1.02	0.50			
MTT_0_	0.99	0.97, 1.03	0.94			

Note. RFS = recurrence-free survival, CI = confidence interval, ER = estrogen receptor, PR = progesterone receptor, HER2 = human epidermal growth factor receptor 2, luminal (ER-positive and/or PR-positive), HER2-enriched (ER and PR-negative, and HER2-positive), and triple-negative (ER-negative, PR-negative, and HER2-negative). NAC = neoadjuvant chemotherapy, CEUS = contrast-enhanced ultrasound, PEAK = peak intensity, TTP = time to peak, RBV = regional blood volume, RBF = regional blood flow, MTT = mean transit time. The quantitative parameters of CEUS before treatment were described as A_0_.

## Data Availability

The datasets used and/or analyzed during the current study are available from the corresponding author on reasonable request.

## Abbreviations

CEUS	contrast-enhanced ultrasound
pCR	pathologic complete response
RFS	recurrence-free survival
NAC	neoadjuvant chemotherapy
PEAK	the maximum intensity
RBV	regional blood volume
TTP	time to peak
RBF	regional blood flow
MTT	mean transit time
ROC	receiver operating characteristic curve
